# Protein Classes Predicted by Molecular Surface Chemical Features: Machine Learning-Assisted Classification of Cytosol and Secreted Proteins

**DOI:** 10.1021/acs.jpcb.4c02461

**Published:** 2024-08-26

**Authors:** Guanghao Hu, Jooa Moon, Tomohiro Hayashi

**Affiliations:** † Department of Materials Science and Engineering, School of Materials Science and Chemical Technology, Tokyo Institute of Technology, 4259 Nagatsuta-cho, Midori-ku, Yokohama-shi, Kanagawa-ken 226-8502, Japan; ‡ The Institute for Solid State Physics, The University of Tokyo, 5-1-5, Kashiwanoha, Kashiwa, Chiba 277-0882, Japan

## Abstract

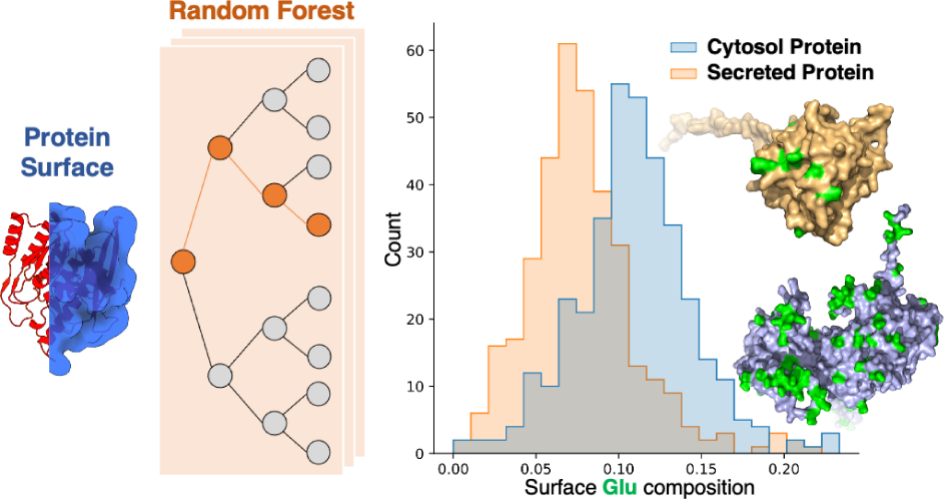

Chemical structures of protein surfaces govern intermolecular interaction, and protein functions include specific molecular recognition, transport, self-assembly, etc. Therefore, the relationship between the chemical structure and protein functions provides insights into the understanding of the mechanism underlying protein functions and developments of new biomaterials. In this study, we analyze protein surface features, including surface amino acid populations and secondary structure ratios, instead of entire sequences as input for the classifier, intending to provide deeper insights into the determination of protein classes (cytosol or secreted). We employed a random forest-based classifier for the prediction of protein locations. Our training and testing data sets consisting of secreted and cytosol proteins were constructed using filtered information from UniProt and 3D structures from AlphaFold. The classifier achieved a testing accuracy of 93.9% with a feature importance ranking and quantitative boundary values for the top three features. We discuss the significance of these features quantitatively and the hidden rules to determine the protein classes (cytosol or secreted).

## 1 Introduction

Proteins have evolved strategies to adapt themselves to their subcellular environments, resulting in optimal functioning within the corresponding environmental context.^1,2^ Consequently, studying protein subcellular localization can reveal the connections between protein properties and functions. The differentiation of protein features in body fluids gains attention, as body fluids serve as the medium for various cellular activities and provide a stable environment for biomaterials to function properly.^3^ Cell membranes divide the body fluid environment into extra- and intracellular parts. Intracellular and extracellular fluids maintain cellular homeostasis and mediate various biological processes. These fluids exhibit distinct compositional differences that facilitate their respective functions.

One primary difference between intra- and extracellular environments lies in the electrostatic potential, which is influenced by the concentration of ions and charged molecules, resulting in a unique electrical environment for each.^4^ The concentration of macromolecules, including proteins, nucleic acids, and carbohydrates, differs significantly between intracellular and extracellular fluids, reflecting the diverse functional requirements inside and outside the cell.^5^ Lastly, protein interactions with membranes and the degrees of molecular crowding vary between intracellular and extracellular environments. Intracellularly, proteins engage with densely packed organelle membranes, participating in signal transduction and vesicle trafficking.^6,7^ In contrast, extracellular proteins interact with the less crowded plasma membrane, contributing to cell adhesion, communication, and nutrient transport.^8,9^ These differences in interaction patterns and membrane crowdedness reflect the diverse functions that proteins serve inside and outside the cell.

Protein structures and surface amino acids strongly correlate with environmental conditions considering specific and nonspecific interactions.^10^ Proteins employ specific amino acid pairings that yield enhanced binding affinities with water molecules, which help prevent nonspecific interactions.^11^ Factors including shape and size, residue propensity, secondary structure exposure, and hydrogen bonding play crucial roles in protein complex formation.^12^ Furthermore, the prevalence of various chemical bonds effectively indicates surface antiadhesion properties.^13^ Prior research has leveraged amino acids as building blocks for modifying biomaterial surfaces, such as in constructing nonfouling self-assembled monolayers^11,14−16^ and polymer films.^17^ These successful applications underscore the connection between surface amino acid characteristics and their functional roles.^14^ Hence, examining surface features contributes to a better understanding of protein behaviors and potential biomaterial designs.

Machine learning has emerged as a powerful tool for managing large-scale data and identifying statistical relationships, showcasing immense potential in uncovering hidden rules from data, particularly in protein research. The continuous updates and publication of protein databases, such as UniProt,^18^ AlphaFold,^19^ Gene Ontology,^20^ and the Protein Data Bank,^21^ provide a wealth of reliable resources like annotations, protein sequences, and structures, significantly enhancing the application of machine learning by allowing the incorporation of various protein features as input for diverse models.^22,23^ Protein-based machine learning models are two major types. One is the deep learning models utilizing protein sequences. These models aim to interpret protein behaviors, such as folding and localization, from the most fundamental genetic features.^24−31^ Though these models gain prediction results with high accuracies, they often need more details of the underlying mechanisms of prediction targets from the aspect of body–body interactions. While researchers have tried to demonstrate results by analyzing the weighting of sequence segments,^22,32^ the findings have limited universality in biomaterial design since applying them to nonprotein entities proves challenging. The other type of model treats proteins as well-folded entities and explains protein characteristics based on established structures. These models analyze protein features such as protein images and surface features to illustrate protein characters from a more macroscopic perspective,^33−38^ enhancing the universality of the results and pushing the outcome more to the aspect of applications.

In this study, we employed machine learning to differentiate between secreted (extracellular) and cytosolic (intracellular) proteins by analyzing protein surface amino acid populations and secondary structure ratios. Additionally, we explained the feature importance by model interpretation and referred to general protein surface properties, aiming to establish a guideline for biomaterial design.

## 2 Methodology

### 2.1 Data Set of Proteins

We constructed our data set using two primary resources: UniProt and AlphaFold. UniProt provides species tags, descriptions of protein subcellular locations, and references to protein 3D structures. We opted for the predicted structural data from AlphaFold rather than the experimental data from PDB due to PDB’s limited data set size and numerous incomplete structures (e.g., major urinary protein 4, PDB ID: 3KFF, AlphaFold ID: AF-P11590-F1). AlphaFold provides more highly accurate predictions for most proteins than the databases, as researchers believe the system has effectively learned the fundamental principles of protein nature.^39,40^ Furthermore, AlphaFold evaluates the confidence of each predicted region using a score, wherein lower scores serve as reliable indicators of intrinsically disordered regions.^21^

Our data set comprises 708 proteins. We initially filtered protein files on UniProt based on the following criteria: (1) a reviewed file; (2) a clear description of the subcellular location, either’secreted’ or’cytosol’; (3) a corresponding 3D structure available on AlphaFold. The protein samples included proteins from humans, mice, and rats to ensure a sufficiently large data set. We tested models at each stage using the new data set to maintain homogeneity across the three data sets. The expanding mouse and rat protein tests demonstrated consistent accuracy, and model performance steadily improved. For each parameter set per training round, we allocated 150 iterations to divide the data set into 70% training data and 30% test data for model training. We balanced secreted and cytosolic proteins in the training data set by separately assigning random states to the protein of each tag to achieve the exact data size (248 cytosol and 248 secreted proteins). The rest of the data set was then used for testing. An extra data set of 106 proteins was also constructed following the same criteria to validate the performance of the best-trained model.

### 2.2 Extract Surface Residues

**Figure 1:**
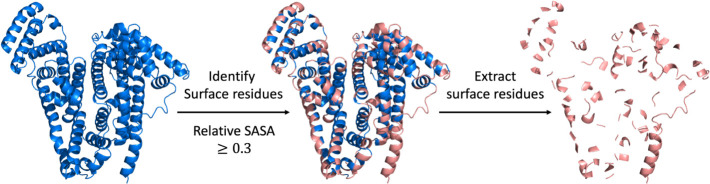
Extract surface residues by the relative solvent-accessible surface area.

We identify surface amino acids by relative solvent-accessible surface area (SASA). Relative SASA is defined as the real SASA divided by the maximum SASA, which refers to the total exposure of the residue of interest when all other residues except for its two neighbors are removed. We first calculated the relative SASA for all residues in the protein. Then, we select residues with a relative SASA higher than or equal to 0.3 as surface residues (Figure 1), and residue information is available in the downstream profiles from the indexes we selected.

### 2.3 Parameters to Describe Surfaces of Protein Molecules

We considered the following factors: (1) surface compositions of the 20 amino acids and groups of amino acids with similar properties; (2) surface populations of functional groups; (3) surface and overall compositions of protein secondary structures; and (4) ratios of surface residues to overall residues.

**Figure 2:**
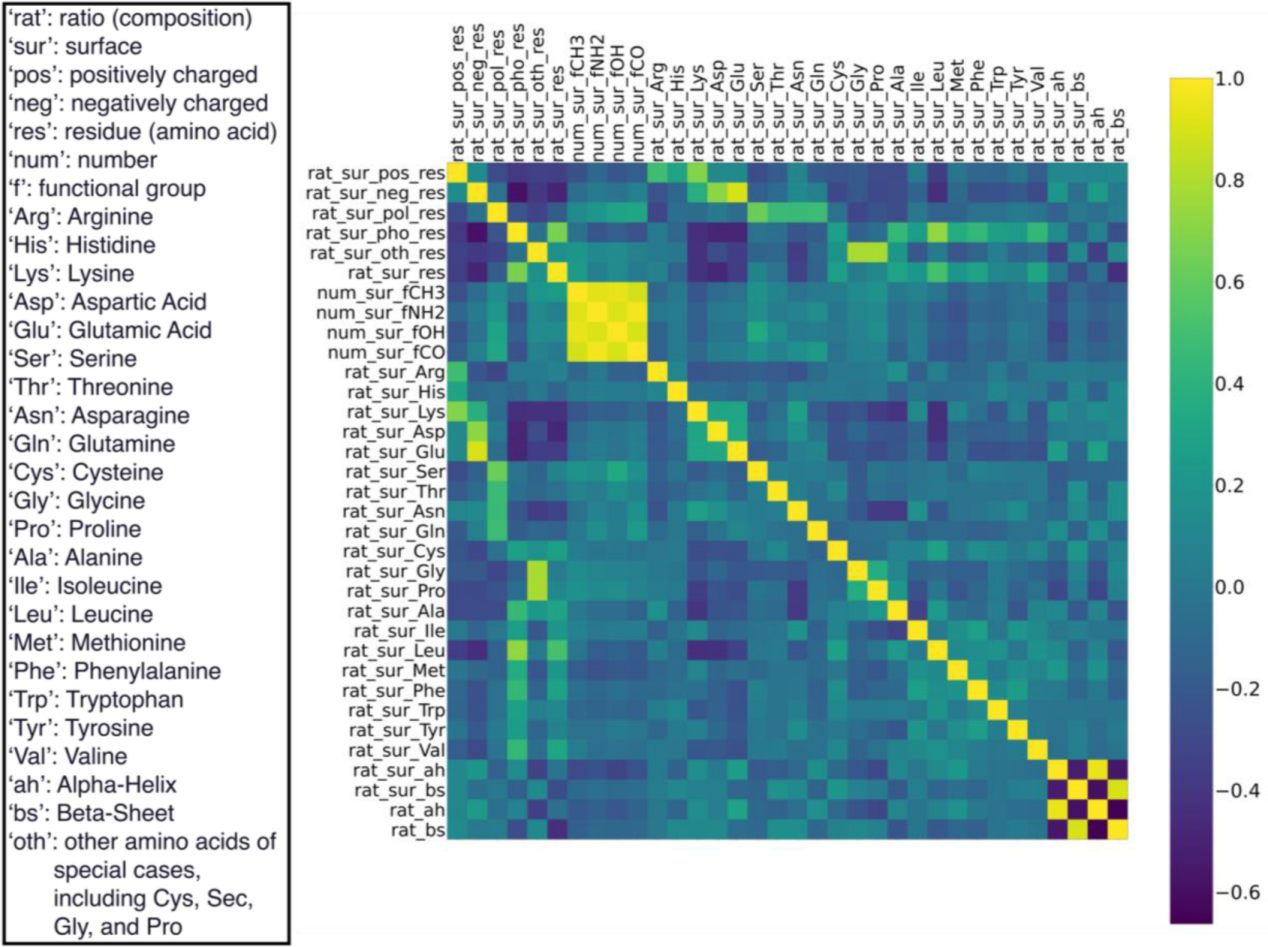
Pearson correlation map of 34 descriptors.

We identified 18 descriptors by checking the Pearson correlation map and the pruning process during model training (Figure 2). The correlation map was employed to identify redundant features. Pairs with a correlation value above 0.85 were selected, and we removed the pairs with a higher cumulative correlation value in each pair. In the pruning process, we did feature importance analysis and deleted features with an importance distribution of less than 2%.

### 2.4 Model Selection

Our purposes require the model to reach a high prediction accuracy while maintaining interpretability, and thus, we selected random forest (RF).

**Figure 3:**
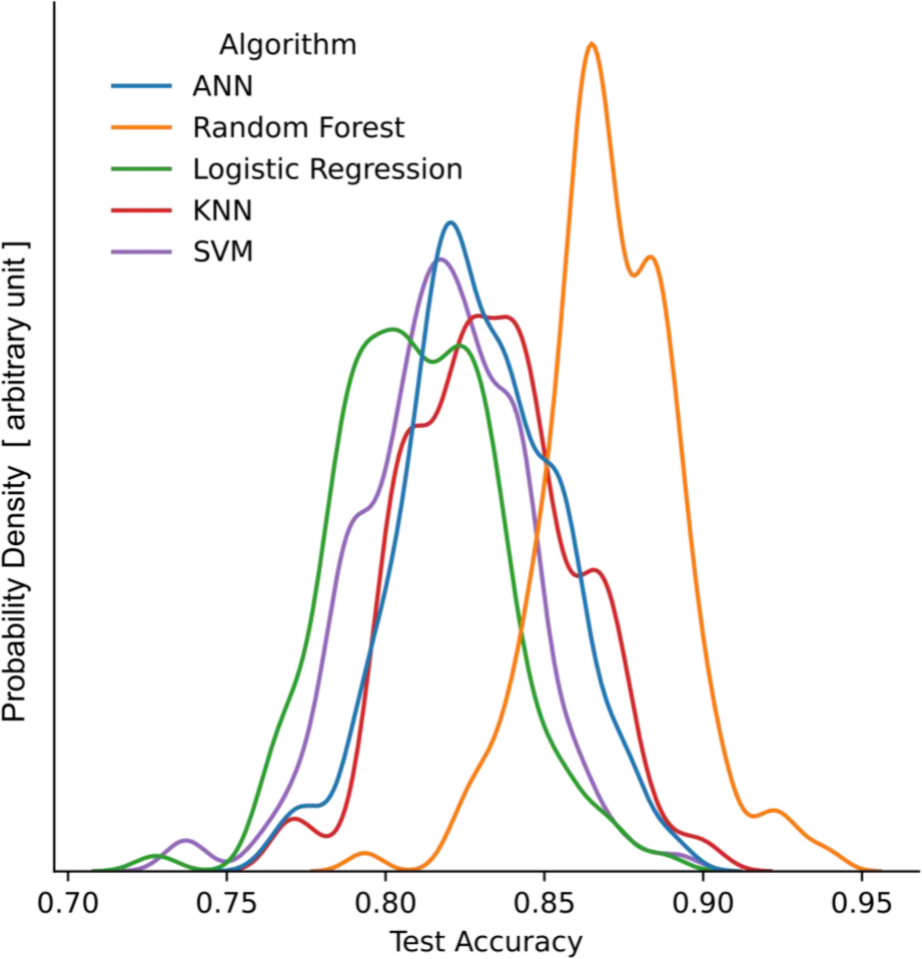
Comparison between the five algorithms. Test accuracy reveals their performances on test data sets, while probability density value indicates the likelihood of model performance to reach the corresponding accuracy.

We compared the performance of RF with four other commonly used algorithms; each has unique features: Artificial neural networks (ANNs) and logistic regression (LR) operate using gradient descent; K-nearest neighbor (KNN) and support vector machines (SVM) rely on distance calculations; the RF algorithm comprises decision trees. We collected performance data for each algorithm by running 150 random states on training-test splitting, and RF demonstrated superior performance (Figure 3). Besides, RF has four main features: (1) based on fully grown decision trees; (2) bootstrapped data for training each tree; (3) majority voting for the final result; (4) assigning random subsets of features for each decision tree.^41,42^ These features make RF advantageous in binary classification problems due to its reduced susceptibility to overfitting and robustness.^43−46^ It also enables feature importance ranking, calculated from the decrease in the Gini index for each feature, which is crucial for identifying predominant features.^42^ The other four algorithms necessitate feature scaling before training, which strictly requires homogeneity between targets and samples from the training data set during the application, while RF does not. This feature allows RF for more flexible applications and direct correlations with the original values of features.

## 3 Results and Discussion

### 3.1 Comparison Between Surface and Global Features

We compared surface and global features across four categories of residue populations: hydrophilic, hydrophobic, positively charged, and negatively charged residues. The category of hydrophilic residues encompasses positively charged residues (Arg, His, and Lys), negatively charged residues (Asp and Glu), and polar but uncharged residues (Ser, The, Asn, and Gln). The hydrophobic residue category includes Ala, Ile, Leu, Met, Phe, Trp, Tyr, and Val.

**Figure 4:**
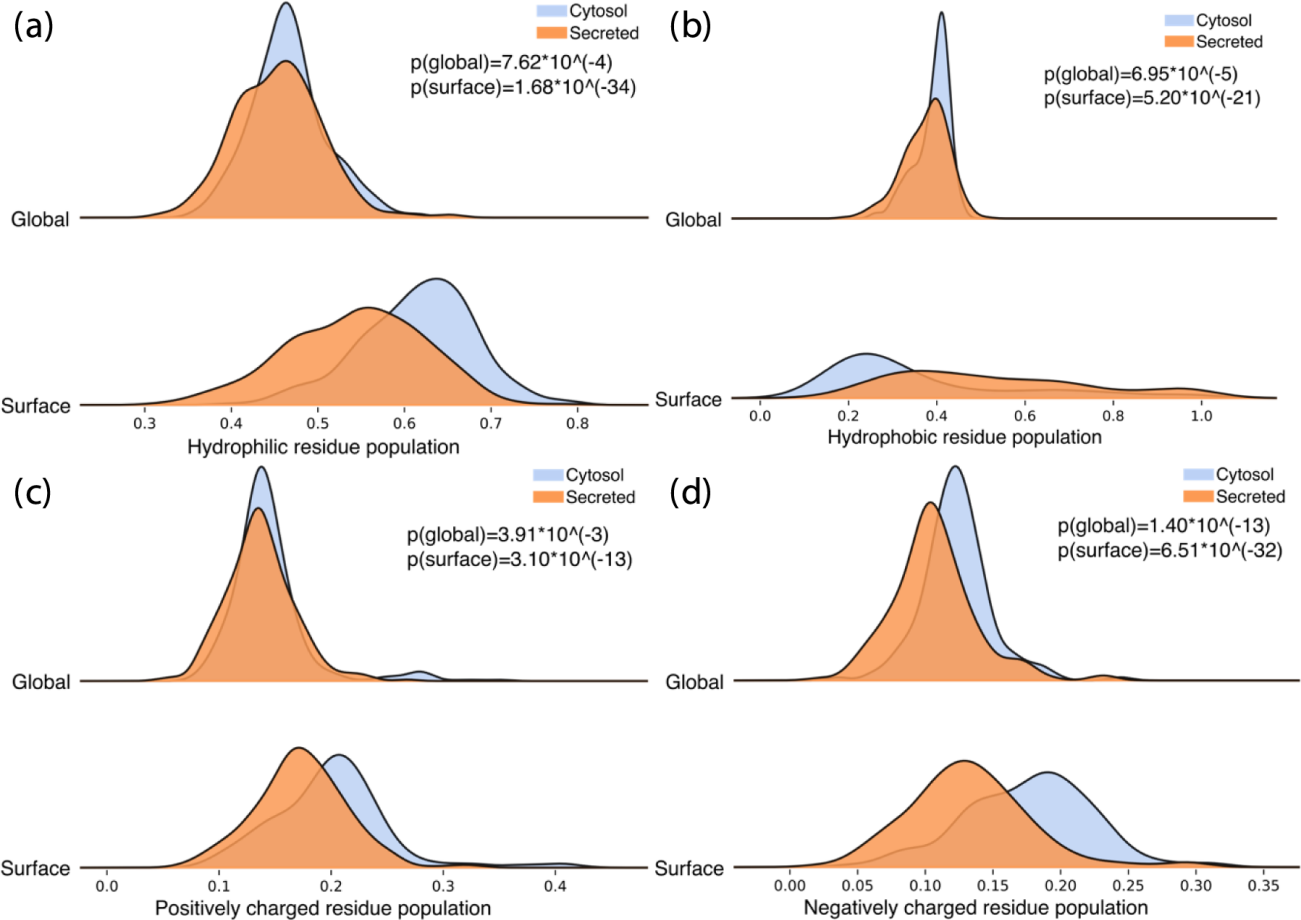
Histograms of the population of four-type residues of secreted (orange) and cytosol (blue) proteins: (a) hydrophilic, (b) hydrophobic, (c) positively charged, and (d) negatively charged. The *p* values are indicated.

Figure 4 shows the population of four collections of residues from secreted and cytosol proteins. Global data generally provide poorer distinctions between secreted and cytosolic proteins, underlining the prominence of surface descriptors over global ones in this binary protein classification problem.

### 3.2 Model Performance

**Figure 5:**
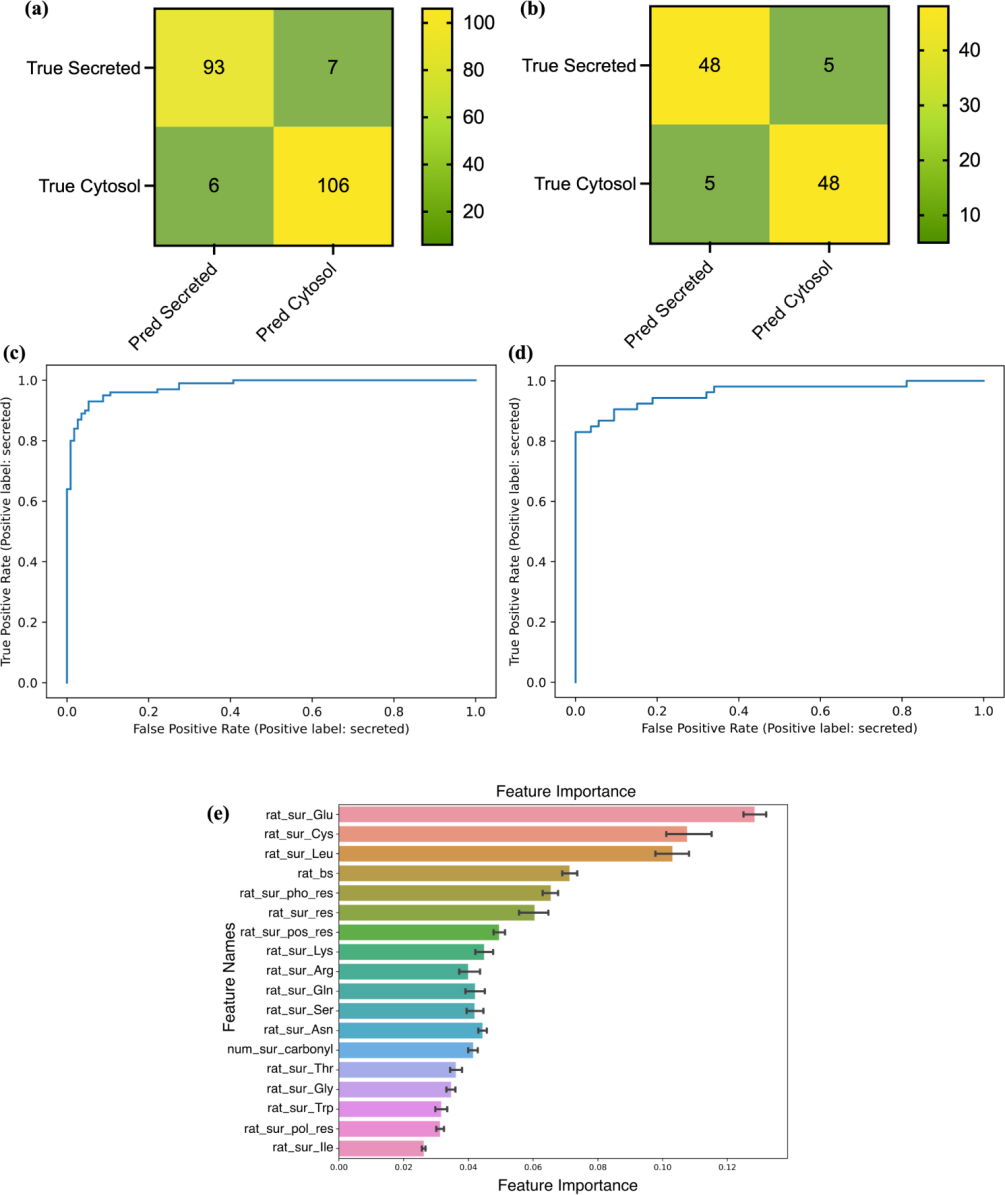
(a) Confusion matrix of the best-trained model on the test data set. (b) Confusion matrix of the model on the validation data set. (c) ROC curve of the model on the test data set. (d) ROC curve of the model on the validation data set. (e) Feature importance ranking from the top-seven RF models.

Confusion matrices and receiver operating characteristic (ROC) curves demonstrate the best-trained model’s capability and robustness to distinguish between positive (secreted proteins) and negative (cytosol proteins) groups (Figure 5a–d). Calculating based on the precision and recall, the model gains an f1 score of 0.935 on the test data set (Table 1). Though it does not reflect the performance on all events, as cytosol and secreted proteins do not hold a definite relationship with negative and positive tags, the f1 score remains as high as 0.906 on the balanced validation data set, indicating the promising performance of the classifier. Besides, the feature importance analysis displayed similar rankings from seven well-trained RF models with an average accuracy of 93.03% (Figure 5b). Among the 18 descriptors, surface compositions of Glu, Cys, and Leu consistently emerged as the leading contributors to the prediction.

**Table 1:** Threshold Values of Surface Compositions of Glu, Cys, and Leu to be Recognized as Secreted Proteins

	precision	recall
test data set	0.939	0.930
validation data set	0.906	0.906

**Figure 6:**
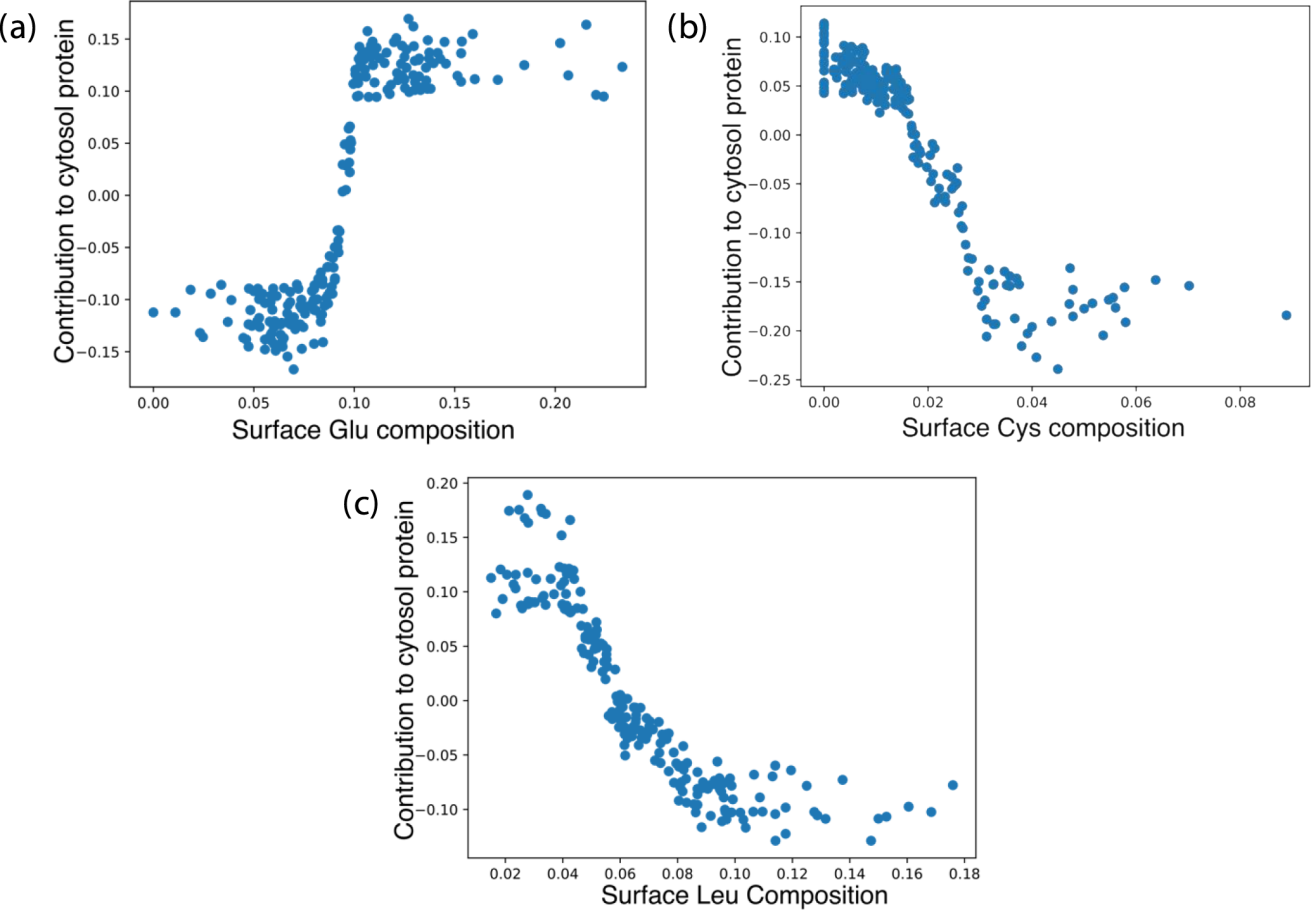
Plots of the contribution of surface (a) Glu, (b) Cys, and (c) Leu compositions toward the chance to be recognized as a cytosol protein in the machine learning.

We discovered relationships between features’ contributions and their values by analyzing the components of several proteins’ probabilities for being classified as secreted or cytosolic proteins. Consequently, we plotted the features’ contributions against their values (Figure 6). Horizontal lines at zero contribution separate data points contributing to secreted and cytosolic proteins, while vertical lines effectively divide the two groups and identify boundary values (Table 2). We found a sigmoid-like distribution pattern of surface Glu, indicating a nonmiscible boundary. Data points from surface Glu to surface Leu gradually aggregate toward the boundary, manifesting the descending importance distribution.

**Table 2:** Threshold Values of Surface Compositions of Glu, Cys, and Leu to be Recognized as Secreted Proteins

	surface Glu	surface Cys	surface Leu
secreted protein	<9.0%	>1.8%	>5.8%

### 3.3 Importance Analysis of Surface Glutamic Acid

Glu and Asp are two amino acids with negatively charged side chains. Their structures only differ by an additional carbon on Glu’s side chain, resulting in similar chemical and physical properties. However, their importance varies significantly. Glu is the top-ranked feature, while Asp was removed during feature pruning due to its low importance. Although they share many similarities, the one-carbon difference influences their preferences for secondary structures in long sequences. Alpha-helices form through hydrogen bonds between the side chains of every first and fourth residue.^47^ While Glu’s structure accommodates this arrangement well, one less carbon in Asp’s structure makes it less favorable for α helix formation.^48,49^

**Figure 7:**
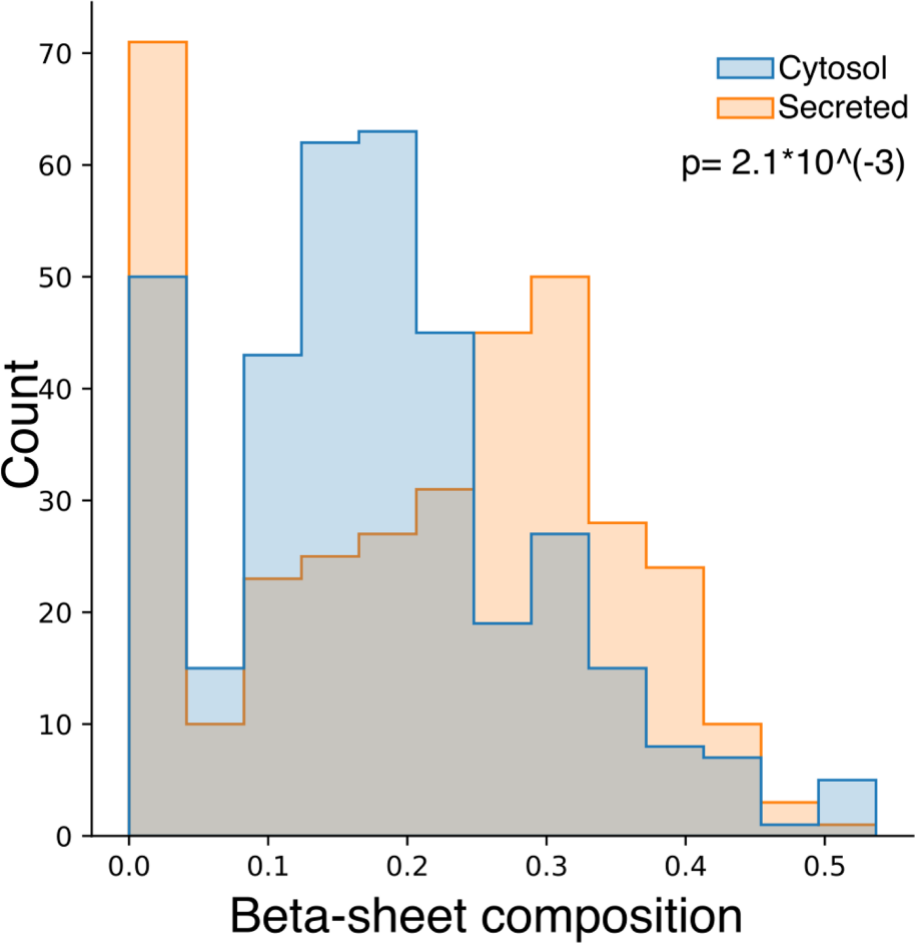
Overall beta-sheet composition distribution of secreted and cytosol proteins.

In this manner, the abundance of surface Glu should correspond to a similar trend in the protein secondary structure. Referring to the correlation map, the alpha-helix and beta-sheet compositions are strongly negatively related. The overall beta-sheet composition plot reveals an expected correlation between the abundance of Glu and deficient surface beta-sheet (Figure 7). Exposing the beta-sheet to the surface promotes aggregation behavior, suggesting that the abundance of Glu could prevent protein aggregation.^50^

Nonetheless, the strong positive correlation between overall and surface secondary structure compositions indicates that alpha-helix and beta-sheet structures do not significantly prefer exposure or burial. Thus, although we have rationalized the relationship between secondary structure and the surface composition of Glu, it may only be a minor contributor to Glu’s importance. The primary importance of Glu’s surface composition arises from its contributions to protein surface hydrophilicity and surface charging state.

**Figure 8:**
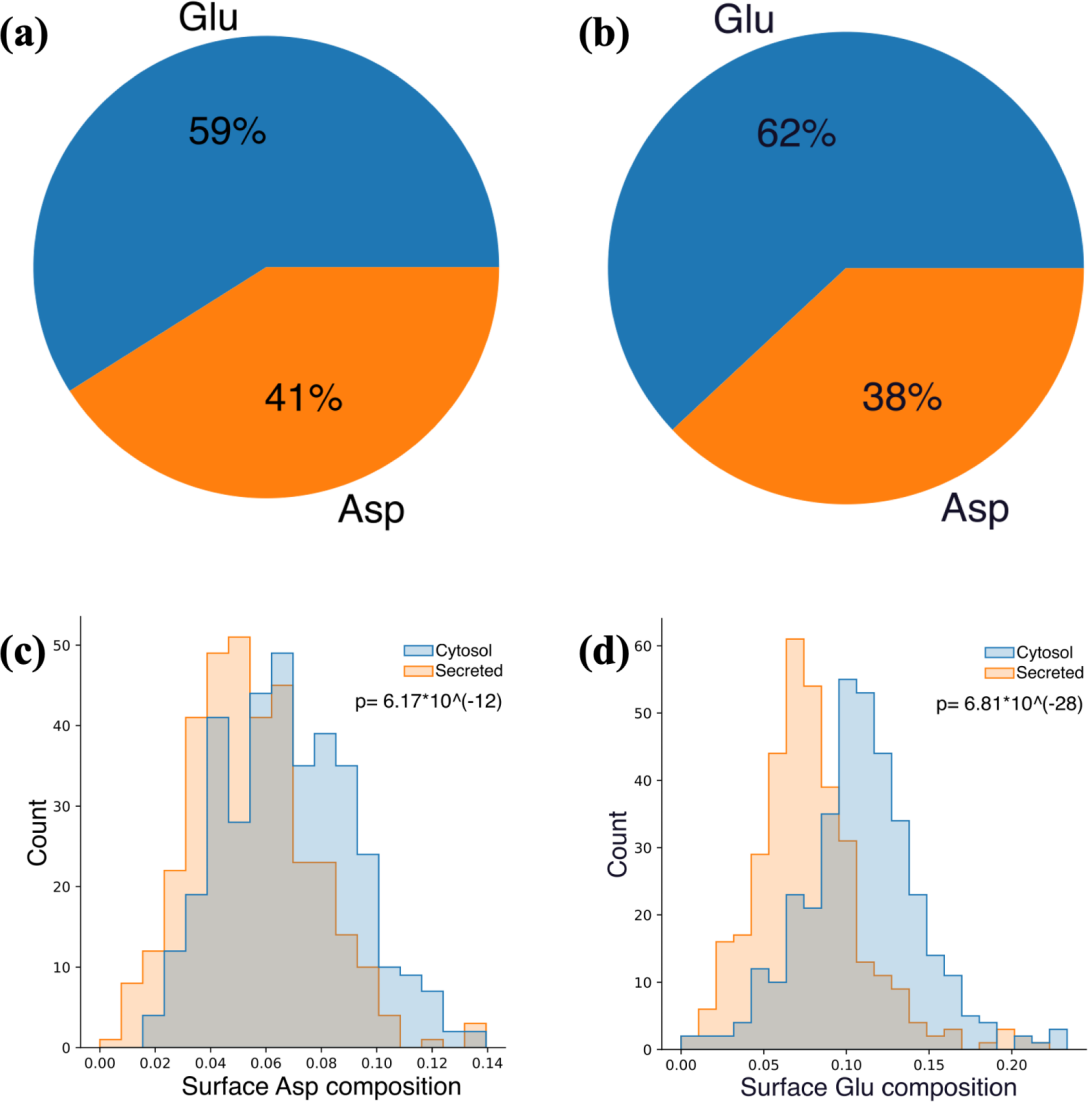
Summary of surface negatively charged residue composition of (a) secreted and (b) cytosol proteins. Surface (c) Asp and (d) Glu distributions of secreted and cytosol proteins.

Glu predominates the population of negatively charged residues for both secreted and cytosolic proteins, where cytosolic proteins have slightly higher Glu compositions (Figure 8a,b). However, such a minor difference still contributes to the discrepancy in protein stability. Glu and Asp differ in their side chain conformational entropy, and substituting Asp with Glu better stabilizes protein conformation from a free energy perspective.^51^ Additionally, previous research reported that an increase in protein denaturation midpoint originates from such substitutions.^52^ Considering the overall surface residues, cytosolic proteins have higher Asp and Glu surface compositions but exhibit a more significant increase in Glu. Therefore, Glu generally represents a larger population on cytosolic protein surfaces, promoting specific interactions in a crowded environment.^53^

**Figure 9:**
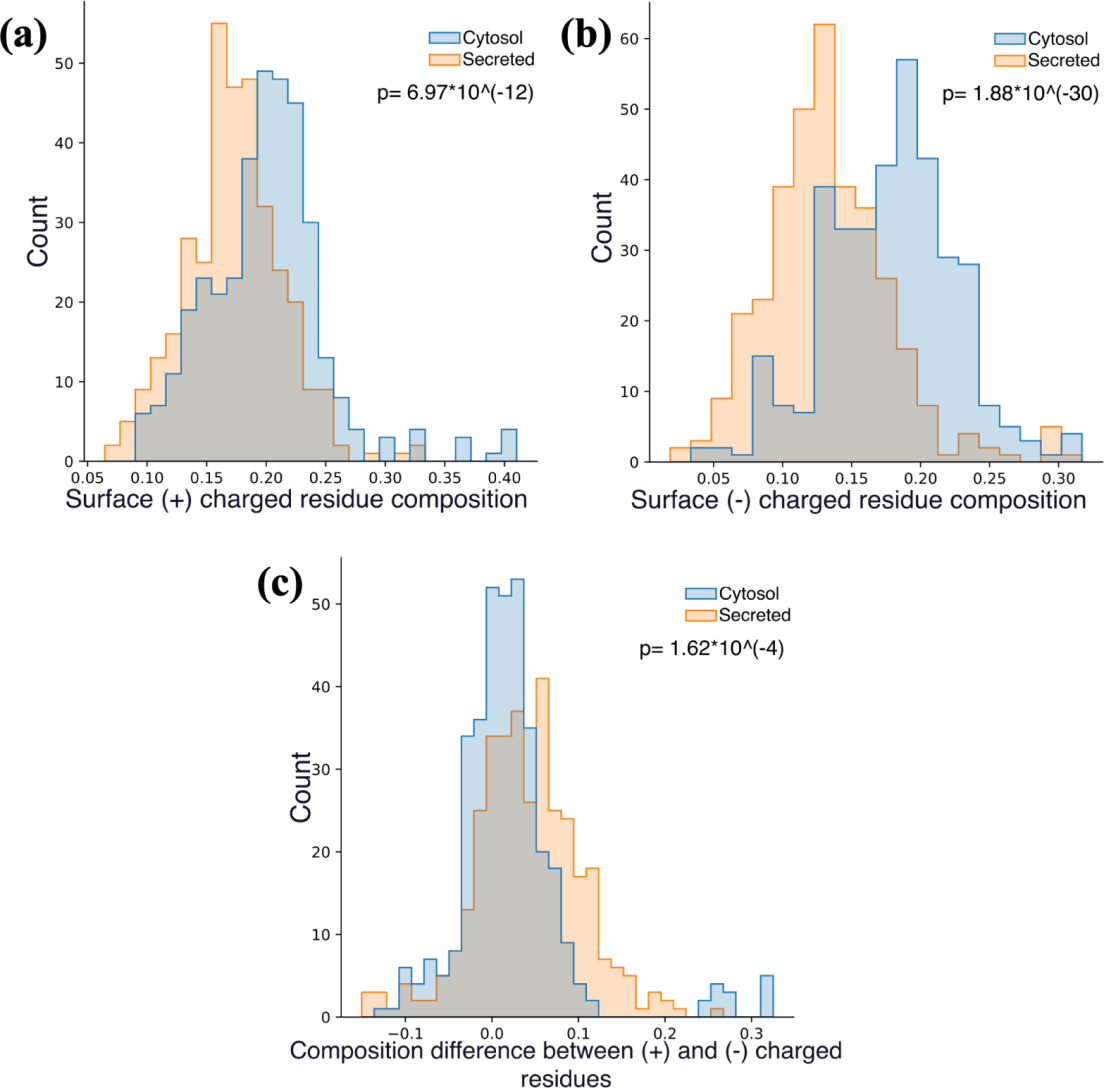
Histogram of secreted and cytosol proteins’ numbers by compositions of (a) surface positively (+) and (b) surface negatively (−) charged residues. (c) Histogram of secreted and cytosol proteins’ numbers by their (surface (+) charged residue composition-surface (−) charged residue composition).

In addition to a greater composition of negatively charged residues on the surface, cytosolic proteins also expose more positively charged residues (Figure 9a,b). This increase in positively charged residues is related to the more negative intracellular electrostatic potential caused by the imbalanced charge separation on the two sides of the cell membrane.^54,55^ However, the increase in surface negatively charged residues is more substantial. Consequently, considering the populational difference between the two types of charged residues (Figure 9c), the overall charges on cytosolic proteins’ surfaces are more neutral. Surface neutrality helps prevent trappings by negatively charged membranes.^56−59^ Thus, the abundance of Glu aids in blocking membrane adhesion and maintaining protein mobility.

**Figure 10:**
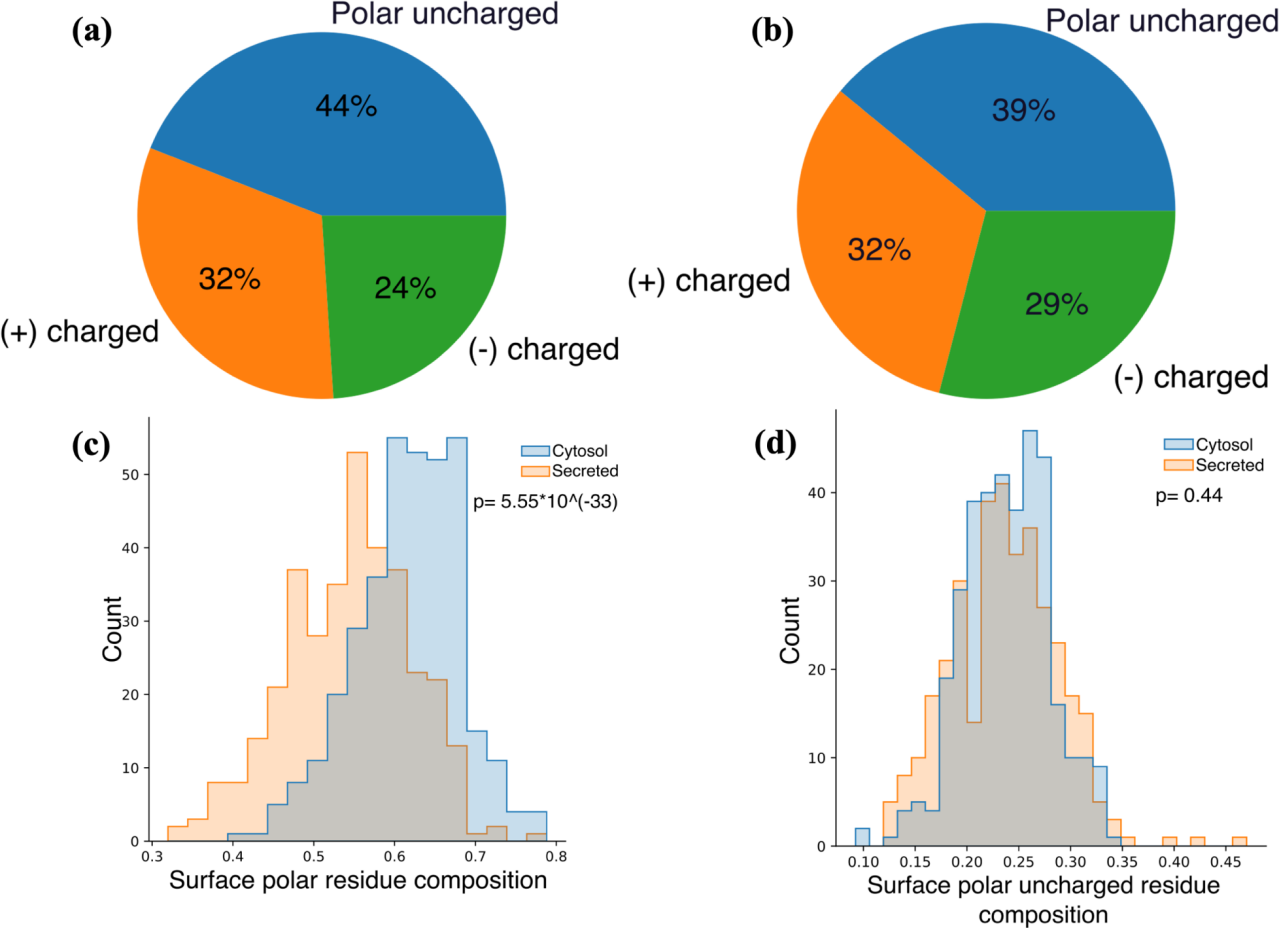
Summary of surface polar residue compositions of (a) secreted and (b) cytosol proteins. Histogram of secreted and cytosol proteins’ numbers by compositions of (c) surface polar and (d) surface nonpolar residues.

We also observed an increase in cytosolic proteins’ surface polar residue composition (Figure 10c). Simultaneously, the composition analysis of surface polar residues reveals that uncharged polar residues have a lower weight in cytosolic proteins, while negatively charged residues have a higher weight (Figure 10a,b). However, the surface compositions of uncharged polar residues remain similar in both secreted and cytosolic proteins (Figure 10d), indicating that the negatively charged residue is the primary factor in the shift in polar residue composition. Such hydrophilicity enables the cytosolic protein surface to interact more effectively with aqueous environments and aids in preventing aggregation under crowded cellular conditions.

### 3.4 Importance Analysis of Surface Cysteine

**Figure 11:**
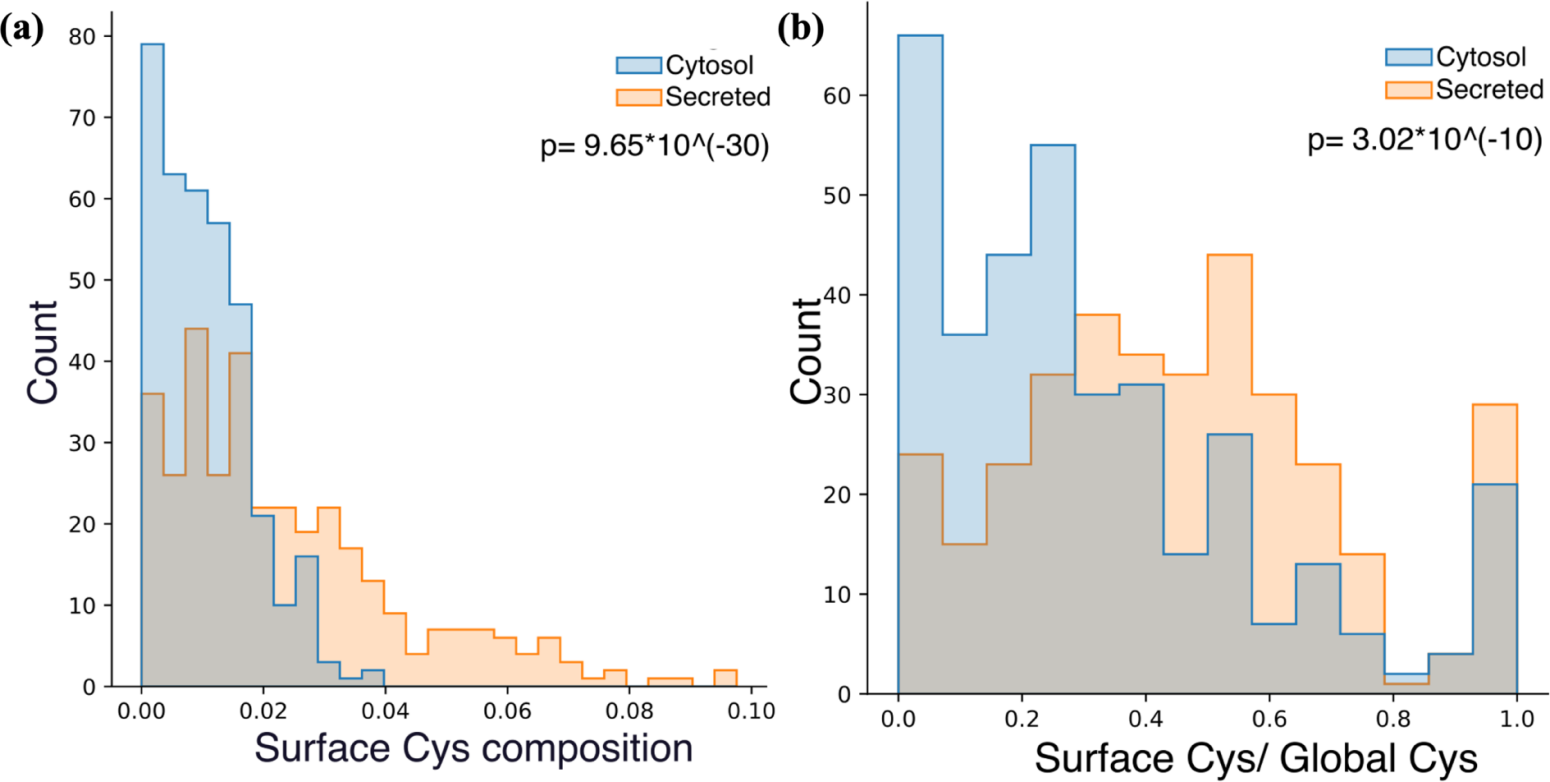
(a) Histogram of secreted and cytosol proteins’ numbers by compositions of surface Cys composition distributions of secreted and cytosol proteins. (b) Histogram of secreted and cytosol proteins’ numbers by the ratio between the numbers of surface Cys and global Cys residues.

Cysteine, characterized by the large atomic radius of sulfur and the low dissociation energy of the S–H bond, is unique among amino acids due to its redox-active function and exceptional nucleophilicity.^60^ Its ionization state is highly susceptible to changes in its immediate chemical milieu. Subtle fluctuations in the reduction potential can induce notable differences in the equilibrium between its dithiol groups and disulfide bonds.^61,62^ Compared to secreted proteins, proteins within the cytosol exhibit a diminished concentration of surface cysteine, as indicated in Figure 11a, and a less pronounced inclination to present cysteine on the surface, as shown in Figure 11b. This observation aligns well with the differing reduction potentials and protein functions inside and outside the cell.

Inside the cell, the chemical environment is reducing, with a potential ranging from −220 to 260 mV, while outside the cell, it is oxidizing at approximately −140 mV.^63^ As such, except in certain hyperthermophilic archaea, intracellular disulfide bonds are scarce and not fundamentally critical for protein structures.^64^ Surface cysteine on cytosol proteins predominantly serves roles in redox-sensitive regulation and protection against reactive oxygen species (ROS), given that it exists predominantly in the reduced form.^65,66^ Conversely, in response to the oxidizing extracellular environment, cysteines form intramolecular disulfide bonds to bolster protein structure integrity and are displayed on the surface to stabilize proteins under harsh environmental conditions.^60,67^ Additionally, surface cysteines aggregate at the active sites of enzymes that catalyze redox processes.^68^ Consequently, the population difference on surface Cys is both protein function- and chemical environment-related one.

### 3.5 Importance Analysis of Surface Leucine

Leu is one of the hydrophobic amino acids, closely resembling Ile and Val. They differ in the length and arrangement of their side chain carbons, which, as mentioned earlier, naturally leads to different preferences for secondary structures. Leu is the second-best preferer for α helix; Val and Ile are the best and second-best preferers, respectively.^49^

**Figure 12:**
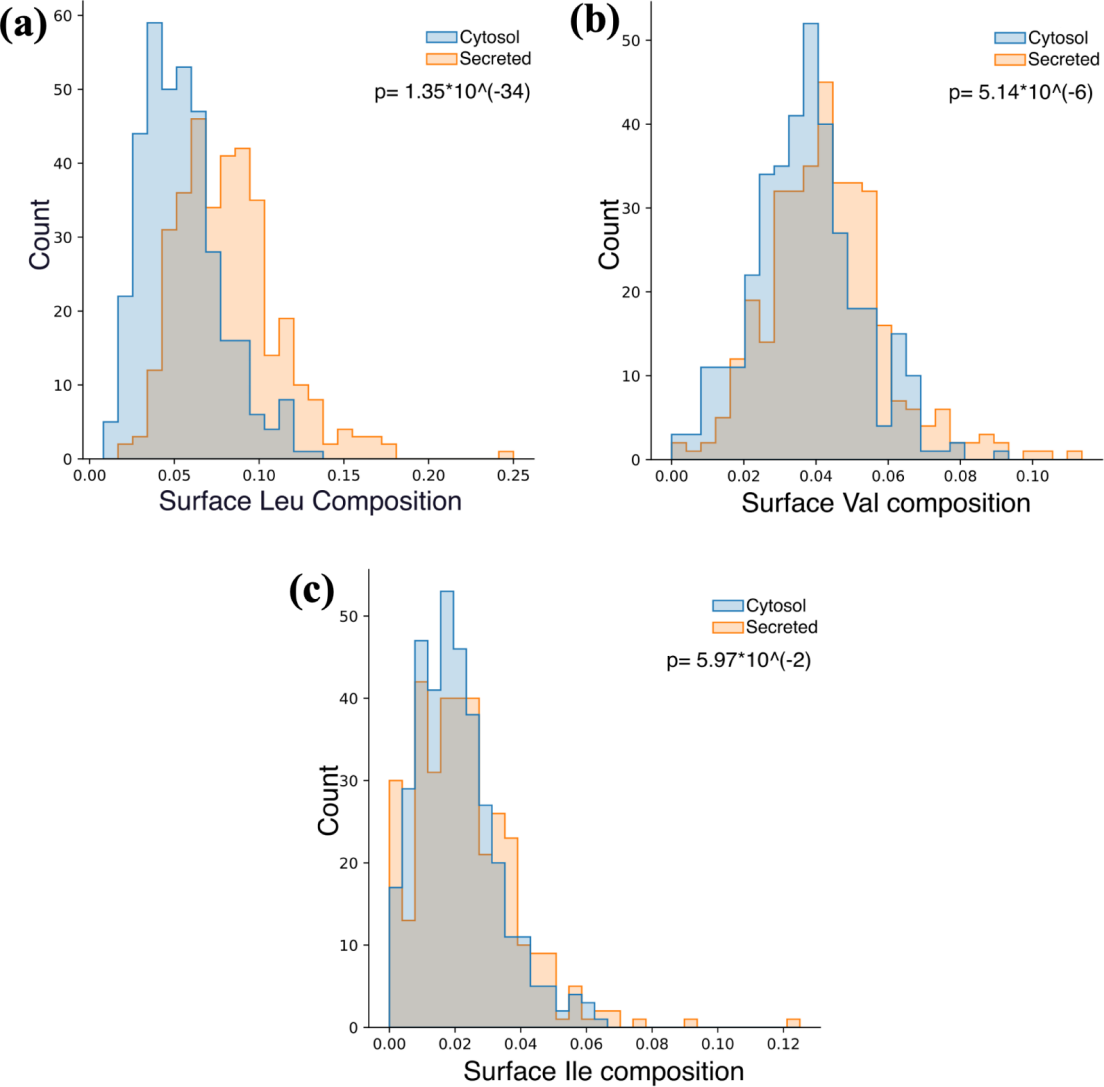
Histogram of secreted and cytosol proteins’ numbers by compositions of (a) surface Leu, (b) surface Val, and (c) surface Ile residues.

Cytosol proteins prefer fewer Leu and Ile residues on the surface, while showing no preference for Val (Figure 12). As mentioned earlier, secondary structural factors are minor contributors to Glu and Asp. The surface compositions of Leu, Val, and Ile further support this idea. Leu and Ile have similar hydrophobicity across various scales, while Val has the weakest hydrophobicity.^69−73^ If secondary structure preference strongly relates to residues’ positions, we expect more significant trends of Val and Ile than Leu to be located in the core. However, the distribution plots do not reveal that.

**Figure 13:**
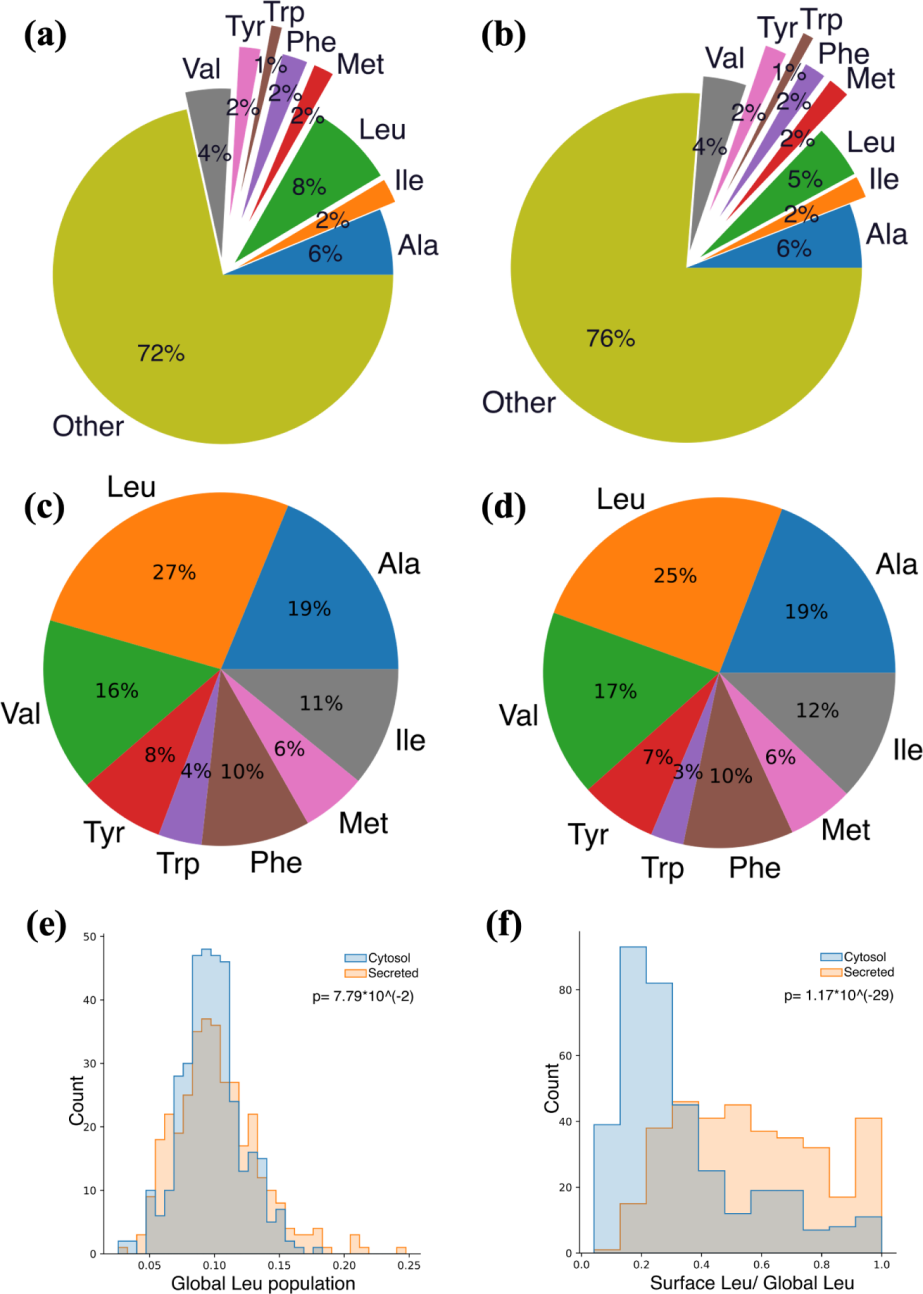
(a) Surface residue composition of hydrophilic (charged and polar) and hydrophobic residues for (a) secreted and (b) cytosol proteins. Compositions of surface hydrophobic residues of (c) secreted and (d) cytosol proteins. Histogram of secreted and cytosol proteins’ numbers by compositions of (e) global and (f) surface Leu residues.

Leu also demonstrates the predominant population in surface and global hydrophobic compositions (Figure 13a,b,d, and e). Compared to secreted proteins, cytosolic proteins have, on average, 3% fewer surface hydrophobic residues, mainly due to the inward shift of Leu from the surface, as Leu’s overall population remains similar for secreted and cytosolic proteins (Figure 13c). The surface-global ratio further illustrates the trend of cytosolic proteins to hide Leu from the surface (Figure 13f), creating a stable hydrophobic core in cytosolic proteins.

### 3.6 Double-Tagged Proteins

**Figure 14:**
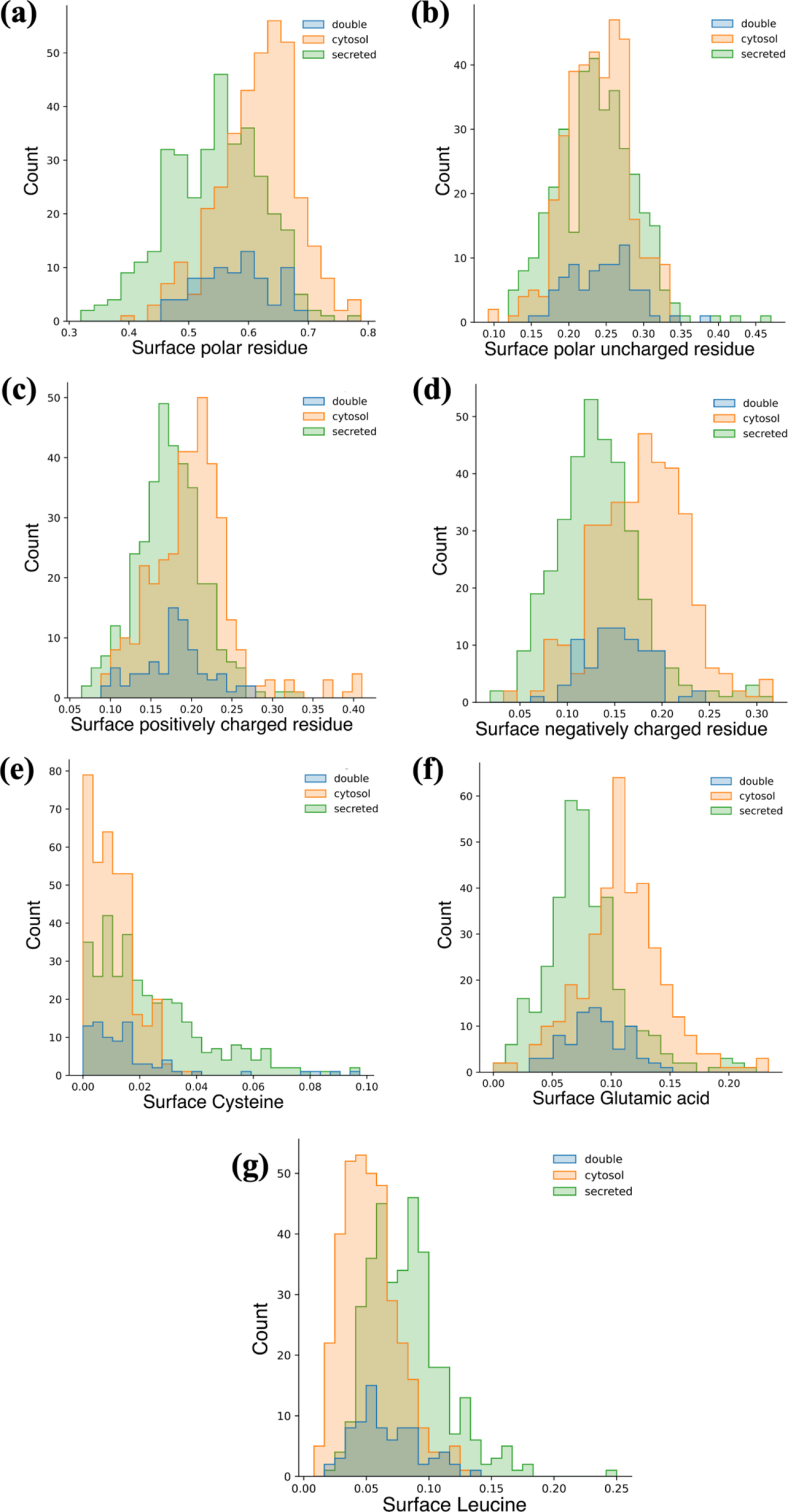
Comparison among secreted, cytosol, and double-tagged proteins on surface features: (a) Surface polar residue composition. (b) Surface polar uncharged residue composition. (c) Surface positively charged residue composition. (d) Surface negatively charged residue composition. (e) Surface cysteine composition. (f) Surface glutamic acid composition. (g) Surface leucine composition.

Besides secreted-only and cytosol-only proteins, some proteins can exist in both environments according to scenarios. Due to the deficiency in data size, we picked 79 double-tagged proteins and compared them with cytosol and secreted proteins on the main collections and most important features. Interestingly, these double-tagged proteins balance out each distinct feature of both kinds (Figure 14). We can roughly tell that their peaks sit between the main peaks of the other two kinds, slightly leaning toward either cytosol or secreted proteins. The comparison reveals that the surface chemical structures of the double-tagged proteins are optimized to accept both intra- and extra-cellular environments.

## 4 Conclusions

Using an interpretable RF model and protein surface features, we successfully classified proteins into secreted or cytosol ones. The model provided quantitative references for protein surface amino acid populations.

The model revealed that surface compositions of glutamic acid, cysteine, and leucine were the three most essential features and provided quantitative threshold values for each of them. Further analysis demonstrated the preferences of both secreted and cytosolic proteins for these three features and quantitative boundary values. Interpretations of the features’ importance explained proteins’ strategies to construct compatible surfaces in two different environments. Glutamic acid gains importance by playing a critical role in tuning the protein surface charge and assisting in the construction of a water barrier through polar interactions. The population of surface cysteines relates to the formation of disulfide bonds and, further, antioxidant aspects. The abundance of cysteine on the surfaces of secreted proteins serves as the stabilization strategy in the oxidizing environments and triggers protein functions. Leucine predominates in the change in surface hydrophobic residue composition. In cytosolic proteins, leucine’s inward shifting constructs a more hydrophilic surface and a more hydrophobic core, which is energetically preferred. Generally, protein stability in a crowded environment requires (1) surface neutrality, (2) exposure of hydrophilic residues while hiding hydrophobic residues, and (3) protection from potential oxidative damage.

The model analysis provides the quantitative threshold values of surface amino acid compositions, offering insights for nonfouling surface design. Besides, the results provide a unique perspective on proteins’ in vivo functioning, contributing to a better understanding of protein nature and potentially assisting in protein engineering endeavors. Though surface feature analysis shows promise in this study, other protein features, such as conformational descriptors and intrinsically disordered sections, still require careful examination. Future work can supplement these aspects and evaluate the rules on various materials and platforms by directly utilizing amino acids or implementing alternative modifications to achieve similar effects.

## Supporting Information Available

Plots showcasing the abilities of other surface features on separating cytosol and secreted proteins, other features include surface-global residue ratio, hydrophobic residues composition, polar residue composition, positively charged residue composition, carbonyl group population, glycine composition, isoleucine composition, lysine composition, serine composition, glutamine composition, arginine composition, asparagine composition, tryptophan composition, and threonine composition, and tables listing proteins included in the analysis (PDF)
