# Supplementary material for: Protein Classes Predicted by Molecular Surface Chemical Features: Machine Learning-Assisted Classification of Cytosol and Secreted Proteins
Source: J Phys Chem B. 2024 Aug 26;128(35):8423–36. doi: 10.1021/acs.jpcb.4c02461 (PMC11382266; doi:10.1021/acs.jpcb.4c02461)
Supplement: Supplementary file 1 — jp4c02461_si_001.pdf [file jp4c02461_si_001.pdf]

[supporting information]

Protein Classes Predicted by Molecular Surface Chemical Features: Machine Learning-Assisted Classification of Cytosol and Secreted Proteins

Guanghao Hu[1], Jooa Moon[1], and Tomohiro Hayashi[1, 2, *]

[1]*Department of Materials Science and Engineering, School of Materials Science and Chemical Technology, Tokyo Institute of Technology, 4259 Nagatsuta-cho, Midori-ku, Yokohama-shi, Kanagawa-ken 226-8502, Japan.*

[2] *The Institute for Solid State Physics, The University of Tokyo, 5-1-5, Kashiwanoha, Kashiwa, Chiba 277-0882, Japan*

[*]corresponding author: tomo@mac.titech.ac.jp

1. Contribution-feature value plots of all features

The main content shows the contribution-feature value plots of the top three features and the beta-sheet ratio, from which we can already tell there is a correlation between the importance a feature gains and how well it separates cytosol- and secreted-contributing

parts in the plot. Here we provide the plots for all the other features, attempting to supplement the understanding of the feature importance ranking.

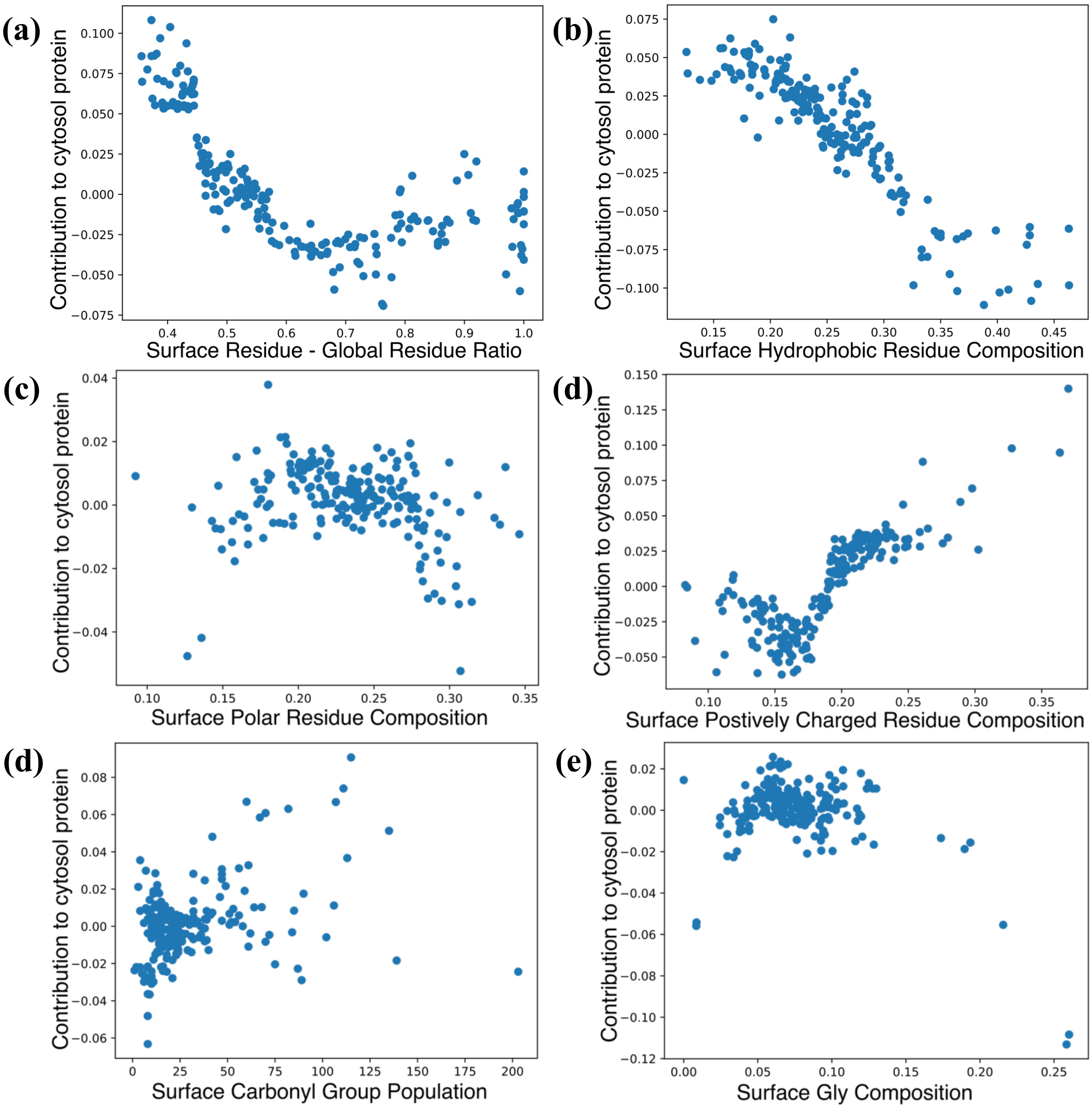

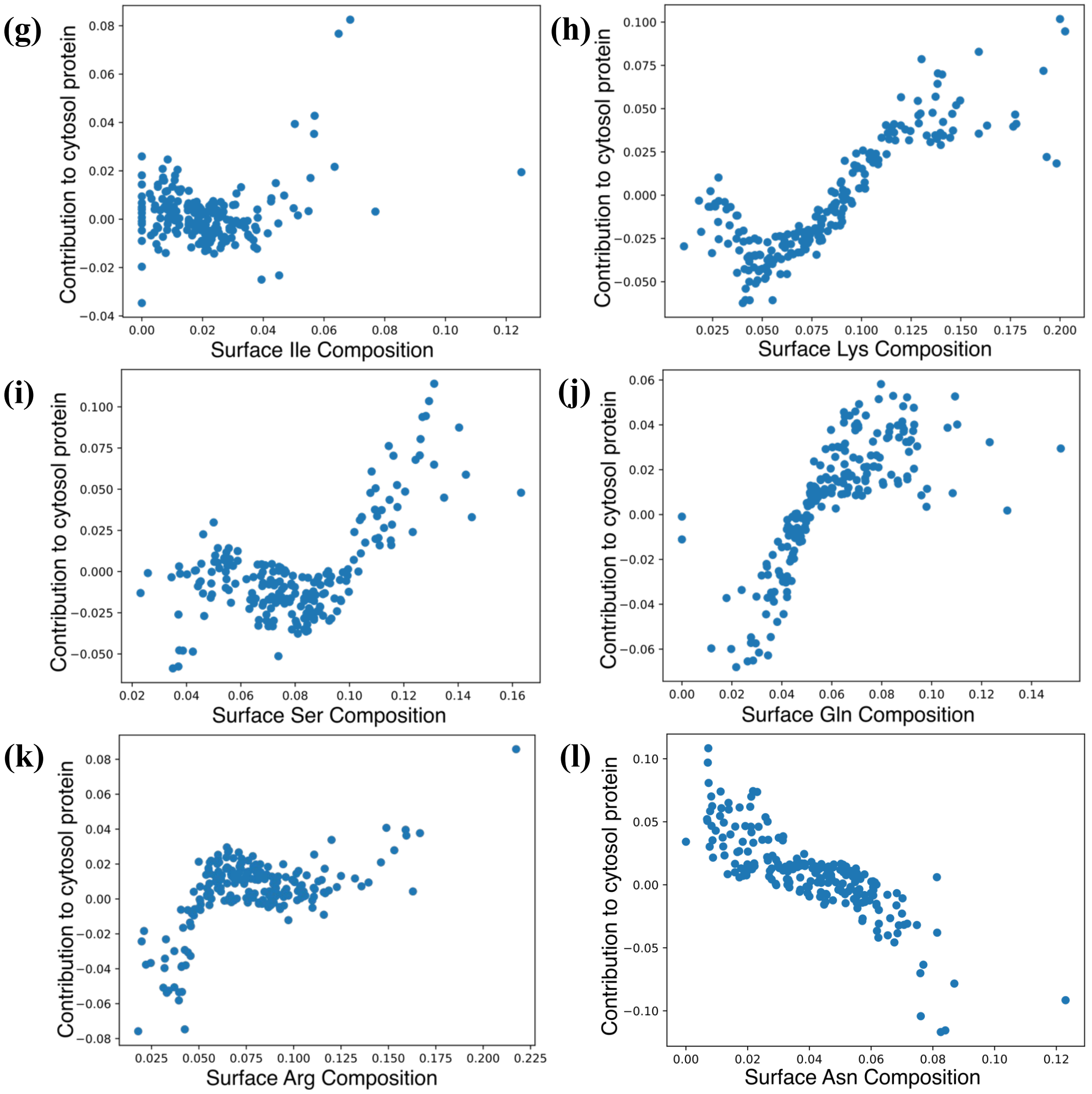

(g)
Contribution to cytosol protein
Surface Ile Composition
(h)
Contribution to cytosol protein
Surface Lys Composition
(i)
Contribution to cytosol protein
Surface Ser Composition
(j)
Contribution to cytosol protein
Surface Gln Composition
(k)
Contribution to cytosol protein
Surface Arg Composition
(l)
Contribution to cytosol protein
Surface Asn Composition

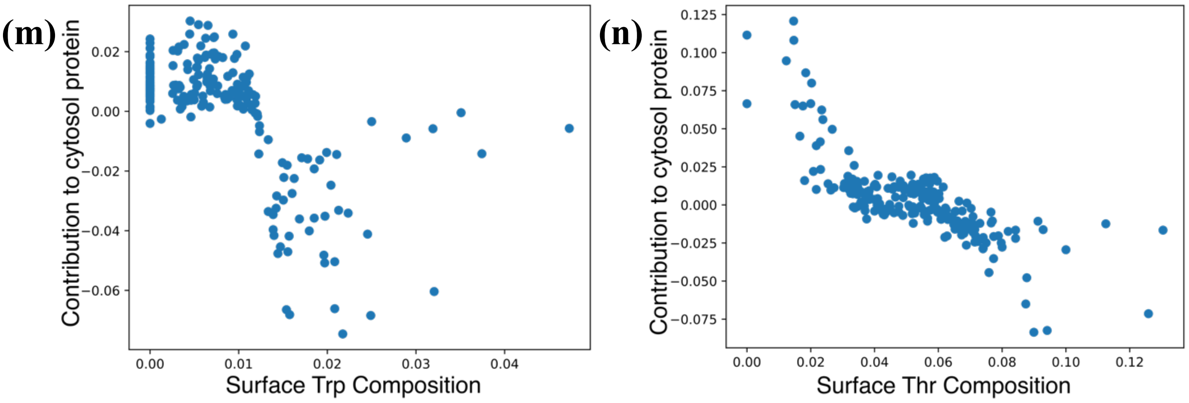

Figure S1. Plots of numerical contribution towards determining a cytosol protein versus feature values of 14 features other than surface Glu, Cys, Leu compositions, and beta-sheet ratio. (a) Ratio between the populations of surface and global residues. (b) Surface hydrophobic residue composition. (c) Surface carbonyl group population. (d) Surface glycine composition. (e) Surface polar residue composition. (f) Surface positively charged residue composition. (g) Surface tryptophan composition. (h) Surface threonine composition. (i) Surface arginine composition. (j) Surface asparagine composition. (k) Surface serine composition. (l) Surface glutamine composition. (m) Surface isoleucine composition. (n) Surface lysine composition.

2. The dataset of proteins

Table S1. ID and the tag of proteins included in the training and testing.

UniProt ID	Tag	UniProt ID	Tag	UniProt ID	Tag	UniProt ID	Tag
P08635	cytosol	Q9CZD0	cytosol	Q91V92	cytosol	Q9D2H2	cytosol
Q5BJZ0	cytosol	P62858	cytosol	Q9JLJ2	cytosol	Q8R1G2	cytosol
Q8BTY1	cytosol	Q7TP52	cytosol	Q91WA6	cytosol	P49888	cytosol
Q5GLZ8	cytosol	P59438	cytosol	Q91X34	cytosol	Q4KLN5	cytosol
Q14032	cytosol	P52566	cytosol	Q9QXG2	cytosol	Q8WYK0	cytosol
Q9CPX5	cytosol	Q96DG6	cytosol	P52895	cytosol	P22061	cytosol
P46953	cytosol	Q9D0I9	cytosol	P39032	cytosol	Q5FVN6	cytosol
F1LQ70	cytosol	Q96G75	cytosol	Q96HC4	cytosol	O00463	cytosol
P63041	cytosol	Q99LS3	cytosol	Q9DBK0	cytosol	P16460	cytosol
Q8K183	cytosol	P81122	cytosol	Q6KAU8	cytosol	Q9JLI6	cytosol
O00214	cytosol	O88673	cytosol	P70191	cytosol	Q86V21	cytosol

UniProt ID	Tag	UniProt ID	Tag	UniProt ID	Tag	UniProt ID	Tag
P51114	cytosol	Q9Y4U1	cytosol	P49189	cytosol	Q8BTG7	cytosol
Q9UFN0	cytosol	D3ZUC6	cytosol	Q04828	cytosol	Q9CR30	cytosol
Q8CA95	cytosol	D3ZVR7	cytosol	Q6AY59	cytosol	Q01066	cytosol
P53396	cytosol	Q68FT9	cytosol	Q6AYG5	cytosol	P14868	cytosol
Q9HAY6	cytosol	O35331	cytosol	P70698	cytosol	Q99M31	cytosol
Q63276	cytosol	Q9D9V3	cytosol	Q3THK7	cytosol	Q96I15	cytosol
Q9QZD5	cytosol	Q8CGY6	cytosol	Q8BGL3	cytosol	Q969F9	cytosol
Q14444	cytosol	Q68FW2	cytosol	Q8R3J5	cytosol	P63040	cytosol
Q8CAB8	cytosol	Q9JMI1	cytosol	P24386	cytosol	P05982	cytosol
Q9Y233	cytosol	Q9D2R0	cytosol	Q6XQN6	cytosol	Q99JY3	cytosol
Q9NTX5	cytosol	P41252	cytosol	Q80VJ4	cytosol	Q7Z739	cytosol
Q9ERD9	cytosol	Q9QY93	cytosol	O43314	cytosol	P35980	cytosol
Q8CI51	cytosol	Q8BU30	cytosol	P70453	cytosol	Q9BUX1	cytosol
P0CG30	cytosol	P28271	cytosol	P14902	cytosol	Q8R5B6	cytosol
Q9H0F6	cytosol	P46844	cytosol	Q13085	cytosol	Q8BHL5	cytosol
P62855	cytosol	A3KGB4	cytosol	Q8BWW4	cytosol	Q8IVU3	cytosol
Q96BM1	cytosol	Q64669	cytosol	P35290	cytosol	A6NDU8	cytosol
Q8BZW8	cytosol	Q0IIM8	cytosol	P05765	cytosol	Q3UHH1	cytosol
P16638	cytosol	Q80U58	cytosol	Q91VC0	cytosol	Q8CFK6	cytosol
Q8IZ69	cytosol	Q9HCR9	cytosol	P49902	cytosol	Q6IMI4	cytosol
Q9CY64	cytosol	P0CC03	cytosol	Q9D5L7	cytosol	Q9NPB8	cytosol
P62856	cytosol	Q9NRN7	cytosol	Q99619	cytosol	Q3V1L4	cytosol
Q6ZU35	cytosol	Q9Y4L5	cytosol	Q8NEM8	cytosol	Q8N9V7	cytosol
Q8CAY6	cytosol	Q78JT3	cytosol	Q5PQN1	cytosol	O60759	cytosol
P28776	cytosol	P53004	cytosol	Q8K349	cytosol	Q9JLJ3	cytosol
P0C090	cytosol	Q9Z1H9	cytosol	Q91XT5	cytosol	Q96B36	cytosol
P51647	cytosol	Q9JHU6	cytosol	Q9JMH3	cytosol	Q149M9	cytosol
Q8BVG4	cytosol	Q8BR90	cytosol	Q9H227	cytosol	P09034	cytosol
Q99NB7	cytosol	Q8N2Y8	cytosol	P46952	cytosol	Q7L5D6	cytosol
Q8N7A1	cytosol	Q6PAV2	cytosol	P09811	cytosol	D3ZMY7	cytosol
Q5RJG7	cytosol	Q9CQF6	cytosol	P23506	cytosol	P28037	cytosol
Q91YQ7	cytosol	P61354	cytosol	Q8CC86	cytosol	O09131	cytosol
Q9DB60	cytosol	Q80U22	cytosol	Q86TX2	cytosol	Q9BRS2	cytosol
Q9Z2L9	cytosol	Q92615	cytosol	Q8R197	cytosol	Q5M819	cytosol
Q9CQR2	cytosol	P49891	cytosol	P47911	cytosol	P48026	cytosol

UniProt ID	Tag	UniProt ID	Tag	UniProt ID	Tag	UniProt ID	Tag
Q8TB72	cytosol	Q5PR69	cytosol	P51556	cytosol	Q9D5U8	cytosol
Q9JL15	cytosol	Q16719	cytosol	Q09M05	cytosol	Q66H61	cytosol
Q96KN8	cytosol	P41562	cytosol	O88844	cytosol	Q3U1T9	cytosol
P0C1Q2	cytosol	Q6P9H5	cytosol	Q5XI22	cytosol	Q9Z339	cytosol
Q9Z224	cytosol	Q60865	cytosol	P21533	cytosol	Q9NY33	cytosol
Q4V7C6	cytosol	O75897	cytosol	Q9HBK9	cytosol	O55137	cytosol
Q01065	cytosol	Q9D1G2	cytosol	P24549	cytosol	Q9NUV9	cytosol
P15559	cytosol	Q6AYB4	cytosol	A6NHX0	cytosol	Q9R0Z7	cytosol
Q66H98	cytosol	Q8IWZ4	cytosol	Q6A009	cytosol	Q96A44	cytosol
Q9QYJ6	cytosol	Q6PD74	cytosol	Q641Z5	cytosol	O94822	cytosol
Q9Y4H2	cytosol	Q80YG3	cytosol	Q0VDF9	cytosol	O70481	cytosol
Q9NV23	cytosol	P70261	cytosol	Q71RC2	cytosol	P52844	cytosol
P54136	cytosol	P62275	cytosol	P78417	cytosol	O75874	cytosol
Q9UPZ3	cytosol	Q922Q2	cytosol	E5FYH1	cytosol	Q99618	cytosol
Q8WTX7	cytosol	Q16773	cytosol	P47964	cytosol	P58137	cytosol
Q2LKV2	cytosol	Q8K3K9	cytosol	A8MTJ3	cytosol	Q9ET01	cytosol
Q06518	cytosol	Q96MI9	cytosol	Q8NBF2	cytosol	Q64627	cytosol
Q8BYK6	cytosol	O88838	cytosol	Q9Y3R4	cytosol	Q8BHT7	cytosol
Q99M54	cytosol	Q9CXF0	cytosol	Q7M753	cytosol	Q61584	cytosol
Q8WTS1	cytosol	Q9R123	cytosol	Q2LKV5	cytosol	P40329	cytosol
O88267	cytosol	Q96M32	cytosol	A2ADY9	cytosol	Q2LKW6	cytosol
Q7TSV4	cytosol	Q6ZQB6	cytosol	Q6XQN1	cytosol	P17516	cytosol
P78330	cytosol	Q69ZJ7	cytosol	Q96M53	cytosol	Q6PCE3	cytosol
Q5S6T3	cytosol	Q9D1H7	cytosol	Q9DBL9	cytosol	Q9D1F4	cytosol
Q8BX80	cytosol	Q8VID6	cytosol	Q5I0L6	cytosol	Q9CWQ8	cytosol
Q4VGL6	cytosol	Q91WU5	cytosol	P62274	cytosol	Q8CAA7	cytosol
Q9JJS6	cytosol	O00764	cytosol	Q9JMD3	cytosol	P00966	cytosol
Q93015	cytosol	Q64559	cytosol	P62268	cytosol	Q0GKD5	cytosol
P61358	cytosol	Q8NAA4	cytosol	Q6A0A2	cytosol	B3STU3	cytosol
Q8R2R3	cytosol	Q3UP24	cytosol	Q91VB4	cytosol	O70166	cytosol
Q9D0C1	cytosol	P47897	cytosol	Q96G03	cytosol	P70712	cytosol
Q5M9G3	cytosol	A0A2Z4LIS9	cytosol	Q8BNV1	cytosol	Q64520	cytosol
P62859	cytosol	B2RYJ4	cytosol	Q63918	cytosol	Q5M877	cytosol
A4D126	cytosol	P37727	cytosol	Q16769	secreted	P21180	secreted
A6H603	cytosol	P29477	cytosol	P29279	secreted	P22692	secreted

UniProt ID	Tag	UniProt ID	Tag	UniProt ID	Tag	UniProt ID	Tag
Q9BWD1	cytosol	Q99KK7	cytosol	Q32ZI2	secreted	P17538	secreted
P49915	cytosol	Q63270	cytosol	P00687	secreted	P21810	secreted
P11497	cytosol	P00352	cytosol	P29459	secreted	O09037	secreted
F1M649	cytosol	Q9NR19	cytosol	O89020	secreted	O35206	secreted
Q8VHT6	cytosol	O75891	cytosol	P04073	secreted	P18406	secreted
Q8R0Y6	cytosol	P07146	secreted	Q6IE47	secreted	Q8N2S1	secreted
Q9ULP0	cytosol	P20863	secreted	O09107	secreted	O70283	secreted
Q91VJ2	cytosol	Q9BQR3	secreted	P04186	secreted	Q96S86	secreted
Q91VY6	cytosol	Q8TER0	secreted	P20033	secreted	Q9BXY4	secreted
Q8VHK0	cytosol	A2AJB7	secreted	P30710	secreted	P09240	secreted
P23743	cytosol	P0DMC4	secreted	P20396	secreted	Q5FVH0	secreted
P22062	cytosol	P09920	secreted	Q9GZV7	secreted	Q7Z4P5	secreted
P06737	cytosol	P01193	secreted	P33581	secreted	P11859	secreted
Q62665	cytosol	P0DML2	secreted	O88207	secreted	P70564	secreted
Q8BML9	cytosol	Q92484	secreted	P32822	secreted	O55183	secreted
Q01064	cytosol	Q9BXR6	secreted	P98173	secreted	Q9BQ16	secreted
Q8BH83	cytosol	Q6MG53	secreted	O89029	secreted	O35565	secreted
O08593	cytosol	Q63751	secreted	O08859	secreted	P09006	secreted
Q5SWU9	cytosol	Q6UXT9	secreted	P20918	secreted	A6NHN0	secreted
P62267	cytosol	P08505	secreted	P08476	secreted	Q07257	secreted
Q9Z223	cytosol	P36953	secreted	Q8CJ42	secreted	P02753	secreted
P26374	cytosol	P04634	secreted	O00622	secreted	Q99MU5	secreted
Q12768	cytosol	Q13253	secreted	Q8N129	secreted	O89093	secreted
F2Z461	cytosol	Q6UX73	secreted	P07724	secreted	E9PV24	secreted
Q96BD6	cytosol	P18761	secreted	P10855	secreted	O75493	secreted
A7E2V4	cytosol	Q99MH3	secreted	P08932	secreted	Q64625	secreted
Q9ULE6	cytosol	P16043	secreted	Q6MZW2	secreted	P04769	secreted
Q96M20	cytosol	P17534	secreted	D4AB34	secreted	O55123	secreted
P35228	cytosol	Q9NP70	secreted	O08712	secreted	O88992	secreted
Q61599	cytosol	Q4G0M1	secreted	Q05028	secreted	P35030	secreted
Q8IWV7	cytosol	B9TQX4	secreted	P19827	secreted	P36955	secreted
P21399	cytosol	P01326	secreted	P08709	secreted	P08620	secreted
Q8NFI3	cytosol	O70497	secreted	Q5GAL7	secreted	P34820	secreted
Q9JM84	cytosol	O15520	secreted	P02762	secreted	Q8N0V4	secreted
P12001	cytosol	P12025	secreted	P20908	secreted	Q5ZQU0	secreted

UniProt ID	Tag	UniProt ID	Tag	UniProt ID	Tag	UniProt ID	Tag
Q5XIG2	secreted	P0C7W2	secreted	P09535	secreted	P18893	secreted
P17897	secreted	P02781	secreted	P35247	secreted	F2Z472	secreted
P12644	secreted	Q9BU40	secreted	P20722	secreted	P06869	secreted
P14106	secreted	P51672	secreted	P11588	secreted	P00796	secreted
Q5JXM2	secreted	Q03591	secreted	P24800	secreted	P18340	secreted
P12850	secreted	O88632	secreted	P03953	secreted	Q63341	secreted
P01586	secreted	Q6ZWJ8	secreted	O08689	secreted	P13562	secreted
P05125	secreted	Q13219	secreted	P58166	secreted	Q5H8A2	secreted
A1A547	secreted	O89101	secreted	Q99674	secreted	Q32ZH5	secreted
P08905	secreted	P01887	secreted	P31722	secreted	P01127	secreted
Q4V7A8	secreted	P21237	secreted	P07758	secreted	P0C172	secreted
P11403	secreted	Q62918	secreted	Q9BXJ2	secreted	P11962	secreted
Q9UNI1	secreted	P04426	secreted	P12246	secreted	P18291	secreted
P56703	secreted	Q9WUW3	secreted	P15656	secreted	P05017	secreted
P21274	secreted	P06879	secreted	P06684	secreted	Q06145	secreted
Q17RW2	secreted	P50229	secreted	P12788	secreted	P0DUB6	secreted
Q6GTS8	secreted	P01572	secreted	Q5FVF9	secreted	P04004	secreted
O09164	secreted	O70460	secreted	A7RDN6	secreted	Q63416	secreted
P11276	secreted	P35444	secreted	Q8N2E2	secreted	O70362	secreted
P00746	secreted	P16301	secreted	O14793	secreted	Q62740	secreted
Q6IE62	secreted	Q5XI62	secreted	P01580	secreted	P23593	secreted
P50609	secreted	O43854	secreted	P01216	secreted	Q0P651	secreted
Q91WW1	secreted	P23943	secreted	P35446	secreted	O55038	secreted
O35468	secreted	Q6NUJ1	secreted	P07759	secreted	P09813	secreted
P12107	secreted	O09043	secreted	P21743	secreted	Q9R1T3	secreted
O88452	secreted	Q15661	secreted	P24592	secreted	Q8WUA8	secreted
Q99P67	secreted	P08226	secreted	P01592	secreted	P08934	secreted
P02772	secreted	P04278	secreted	O35622	secreted	P81278	secreted
G3V8D4	secreted	P06728	secreted	O09051	secreted	Q8WWU7	secreted
P04187	secreted	P01023	secreted	A8K2U0	secreted	Q9UHI8	secreted
O35417	secreted	E1U8D0	secreted	Q9NQ38	secreted	Q8N6G6	secreted
P00688	secreted	O88273	secreted	P00683	secreted	P05408	secreted
P01325	secreted	Q99MF5	secreted	O95156	secreted	O88783	secreted
P01346	secreted	P04401	secreted	P01027	secreted	P16294	secreted
O54908	secreted	Q96KN2	secreted	Q8VBX1	secreted	Q15848	secreted

UniProt ID	Tag	UniProt ID	Tag	UniProt ID	Tag	UniProt ID	Tag
Q8NI99	secreted	O70513	secreted	A6X935	secreted	A8TX70	secreted
O54775	secreted	P01242	secreted	P20109	secreted	O35568	secreted
Q5VTL7	secreted	Q6GPI1	secreted	P07490	secreted	Q5VYY2	secreted
P12961	secreted	O35314	secreted	P11590	secreted	Q7Z304	secreted
P11589	secreted	O75094	secreted	P11680	secreted	P08721	secreted
P45452	secreted	P10745	secreted	P04202	secreted	P50230	secreted
P04938	secreted	P01008	secreted	O35608	secreted	P02780	secreted
Q6MG84	secreted	O88430	secreted	Q8IUA0	secreted	C9JL84	secreted
O55233	secreted	P07092	secreted	P10148	secreted	Q6PEZ8	secreted
P18065	secreted	P04939	secreted	P10889	secreted	Q05702	secreted
P52823	secreted	O95972	secreted	Q68FP3	secreted	P11477	secreted
O08665	secreted	Q9UKP5	secreted	P04351	secreted	Q8IVN8	secreted
O94769	secreted	O88947	secreted	P17125	secreted	P12968	secreted
O08677	secreted	O75636	secreted	P02650	secreted	P14847	secreted
Q92563	secreted	Q93098	secreted	P36980	secreted	O89098	secreted
Q62635	secreted	P02749	secreted	O70326	secreted	O88188	secreted
Q62713	secreted	Q7Z5J1	secreted	Q96SM3	secreted	P12032	secreted
Q9UKR3	secreted	P12804	secreted	O95407	secreted	O08746	secreted
P07750	secreted	Q99985	secreted	P07321	secreted	O95388	secreted
P17515	secreted	Q14624	secreted	P18075	secreted	O75095	secreted
P06767	secreted	Q9WUK5	secreted	P01587	secreted	P12645	secreted
P14095	secreted	Q5RJL6	secreted	O15041	secreted	P06303	secreted
P11214	secreted	P02743	secreted	Q5GFL6	secreted	P09056	secreted
P08121	secreted	P11685	secreted	P14097	secreted	A2AX52	secreted
Q9P0W0	secreted	Q8NEV9	secreted	P06880	secreted	P21275	secreted
P09036	secreted	P10923	secreted	P03971	secreted	O35903	secreted

Table S2.ID and the tag of proteins included in the validation.

UniProt ID	Tag	UniProt ID	Tag	UniProt ID	Tag	UniProt ID	Tag
P35754	cytosol	P00558	cytosol	P31371	secreted	P00738	secreted
P37108	cytosol	P13489	cytosol	P34096	secreted	P00740	secreted
P40227	cytosol	P13693	cytosol	O15123	secreted	P00748	secreted
P40429	cytosol	P27348	cytosol	O75718	secreted	P00749	secreted
P40925	cytosol	P35998	cytosol	O76076	secreted	P01011	secreted
P42766	cytosol	P46776	cytosol	O95393	secreted	P01033	secreted
P52565	cytosol	P47914	cytosol	O95841	secreted	P01037	secreted
P60866	cytosol	P52209	cytosol	P00734	secreted	P01042	secreted
P61254	cytosol	P62424	cytosol	P41221	secreted	P01591	secreted
P61981	cytosol	P62888	cytosol	P55000	secreted	P02647	secreted
P62269	cytosol	Q15102	cytosol	P56704	secreted	P02649	secreted
P62829	cytosol	Q15185	cytosol	P61626	secreted	P02790	secreted
P62899	cytosol	Q16543	cytosol	Q00604	secreted	P05019	secreted
P62910	cytosol	Q9Y3U8	cytosol	Q08629	secreted	P05154	secreted
P62917	cytosol	P30043	cytosol	Q12805	secreted	P05305	secreted
P78371	cytosol	P30086	cytosol	Q14393	secreted	P09228	secreted
P83731	cytosol	P35908	cytosol	Q15389	secreted	P11150	secreted
P84098	cytosol	P36578	cytosol	Q2WEN9	secreted	P11597	secreted
Q02543	cytosol	P46779	cytosol	Q6FHJ7	secreted	P12724	secreted
Q99832	cytosol	P49368	cytosol	Q6ZMJ4	secreted	P14138	secreted
P00338	cytosol	P50453	cytosol	Q8N474	secreted	P17936	secreted
P08729	cytosol	P50502	cytosol	Q8N8U9	secreted	P20142	secreted
P18077	cytosol	P50914	cytosol	Q8WX77	secreted	P20800	secreted
P18621	cytosol	P60174	cytosol	Q96DR5	secreted	P24387	secreted
P25786	cytosol	P61313	cytosol	Q96LR4	secreted	P25311	secreted
P26373	cytosol	Q06830	cytosol	Q9BTY2	secreted		
Q9UKY7	cytosol	M5A8F1	secreted	P26022	secreted		

3. Parameters of models

Table S3. Parameter of models.

Algorithm	Parameter
Random Forest	n_estimators=4000, max_features='auto'
Artificial Neural Network	nn.Sequential(<br>nn.Linear(18,9),<br>nn.ReLu(),<br>nn.Linear(9,1),<br>nn.Sigmoid())<br>criteria=nn.BCELoos(), ietration=10000,<br>learning_rate=0.02,<br>optimizer=torch.optim.SGD(...)
Logistic Regression	penalty='l2', dual=False, tol=1e-4, C=1,<br>fit_intercept=True, intercept_scaling=1,<br>solver='lbfgs'
K-Nearest Neightbor	n_neighbors=2, weights='uniform',<br>algorithm='auto', leaf-size='30', p=2,<br>metric='minkowski'
Support Vector Machine	C=1, kernel='linear', degree=3,<br>gamma='scale', coef=0, shrinking=True,<br>probability='False', tol=1e-3,<br>cache_szie=200, verbose=False,<br>max_inter=-1,<br>decision_fucntion_shape='ovr'
